# Data Mining, Network Pharmacology, and Molecular Docking Explore the Effects of Core Traditional Chinese Medicine Prescriptions in Patients with Rectal Cancer and Qi and Blood Deficiency Syndrome

**DOI:** 10.1155/2021/1353674

**Published:** 2021-08-02

**Authors:** Shiyu Ma, Lin Zheng, Lan Zheng, Xiaolan Bian

**Affiliations:** ^1^Department of Pharmacy, Ruijin Hospital Affiliated to Shanghai Jiaotong University School of Medicine, Shanghai, China; ^2^Department of Bone and Joint Surgery, Shanghai GuangHua Hospital of Integrated Traditional Chinese and Western Medicine, Shanghai, China; ^3^Department of Traditional Chinese Medicine, Ruijin Hospital Affiliated to Shanghai Jiaotong University School of Medicine, Shanghai, China; ^4^Department of Pharmacy, Ruijin Hospital Lu Wan Branch, Shanghai Jiaotong University School of Medicine, Shanghai, China

## Abstract

**Background:**

“Zheng” (syndrome) is the basic unit and the basis of traditional Chinese medicine (TCM) treatment. In clinical practice, we have been able to improve the survival time and quality of life for patients with rectal cancer through the treatment of “FuZhengXiaoJi” (strengthening the Qi and reducing accumulation).

**Purpose:**

In this study, we elucidated the core prescriptions for patients with rectal cancer and Qi and blood deficiency syndrome, and we explored the potential mechanisms of the prescriptions using an integrated strategy that coupled data mining with network pharmacology.

**Methods:**

A Bron–Kerbosch (BK) algorithm was applied to find the core prescriptions. The active ingredients, targets, activated signaling pathways, and biological functions of core prescriptions were analyzed using network pharmacology and directly associated proteins were docked using molecular docking technology to elucidate the multicomponent, multitarget, and inter-related components associated with TCM systematically.

**Results:**

Data mining identified 3 core prescriptions, and most of the herbs consisted of “FuZhengXiaoJi” Fang. Network pharmacology identified 15 high-degree active ingredients among the 3 core prescriptions and 16 high-degree hub genes linked with both rectal cancer and the 3 core prescriptions. Additional Gene Ontology and Kyoto Encyclopedia of Genes and Genomes enrichment analyses of these 16 targets showed that the most significant pathways were MAPK, interleukin-17, tumor necrosis factor (TNF), and vascular endothelial growth factor (VEGF) pathways. From the 16 genes, TGFB1, IL1B, IL10, IL6, PTGS2, and PPARG closely interacted with the tumor microenvironment, and PPARG, MYC, and ERBB2 were closely linked to survival. In molecular docking, quercetin, kaempferol, and lauric acid showed good binding energy to each target.

**Conclusion:**

Data mining, network pharmacology, and molecular docking may help identify core prescriptions, high-degree ingredients, and high-degree hub genes to apply to diseases and treatments. Furthermore, these studies may help discover hub genes that affect the tumor microenvironment and survival. The combination of these tools may help elucidate the relationship between herbs acting on “Zheng” (syndrome) and diseases, thus expanding the understanding of TCM mechanisms.

## 1. Introduction

Rectal cancer is a common type of colorectal cancer (CRC). CRC is the second and fourth most common malignant tumor in the world and in China, respectively (approximately 37.6 per 100,000 in 2015) [1, 2], and has a mortality rate of 19.1 per 100,000 [2]. CRC has become a substantial burden in China, particularly in the more developed provinces [3]. Unfortunately, more than 50% of patients with CRC are diagnosed at an advanced stage [4]; according to previous reports, 20%–50% of patients with rectal cancer will eventually develop metastatic disease [5]. At an advanced stage, the 5-year survival rate of CRC is less than 14%.

Effective treatments for CRC include a combination of surgery, chemotherapy, radiation therapy, targeted therapy, and immunotherapy therapy. However, chemotherapeutics have known limitations, such as their substantial adverse effects, including gastrointestinal reactions, mucositis, bone marrow suppression, neurotoxicity, abnormal liver or kidney function, febrile neutropenia, and fatigue [6]. The search for more effective alternative agents with lower toxicity that are able to inhibit the tumor's metastatic potential is essential [7, 8].

Traditional Chinese medicine (TCM) has been used to treat CRC for more than 6,000 years with some degree of success [9]. Good results, including better efficiency and lower toxicity, prolonged survival periods, and improved quality of life, have also been achieved by combinations of TCM with chemotherapy, radiation therapy, targeted therapy, and immunotherapy for the treatment of advanced CRC [10]. Based on a meta-analysis, five herbs, including GanCao (*glycyrrhizae radix et rhizoma*), BaiZhu (*atractylodis macrocephalae rhizoma*), HuangQi (*astragali radix*), DangShen (*codonopsis radix*), and ChenPi (*citri reticulatae pericarpium*), were identified as associated with significant reductions in chemotherapy-induced gastrointestinal (CIGI) toxicity (nausea and vomiting, diarrhea, abdominal pain, abdominal bleeding, ulcerative lesions along the gastrointestinal tract, etc.) [11]. In addition, Si-Jun-Zi decoction [12], which contains GanCao, BaiZhu, FuLing (*poria*), and RenShen (*ginseng radix et rhizoma*), has alleviated CIGI toxicity, including nausea, vomiting, and diarrhea, better than chemotherapy alone.

Some herbs increase the effects of radiotherapy and chemotherapy and prevent metastasis. BaiHuaSheSheCao (*Hedyotis diffusa Willd*) inhibits VEGF-C-mediated lymphangiogenesis in CRC through multiple signaling pathways [13]. Dang-Gui-Bu-Xue decoction (DangGui and HuangQi) induces autophagy-associated cell death in CT26 cells and may have potential as a chemotherapy or radiotherapy sensitizer in CRC treatment [14].

Data mining is a combination of machine learning, artificial intelligence, and other technologies [15]. It estimates correlations by analyzing large amounts of data and extracting hidden relationships and information. In recent years, data mining technology has been extensively applied to understand the activities of TCM treatments better. Network pharmacology is a new technology that obtains information by systematic observation of the intervention and the influence of drugs on the disease from a holistic perspective; it has been successfully and widely applied in many research fields related to TCM [16, 17]. The network pharmacology approach has clear advantages compared with conventional methods in deepening the understanding of comprehensive mechanisms [18].

**Figure 1:**
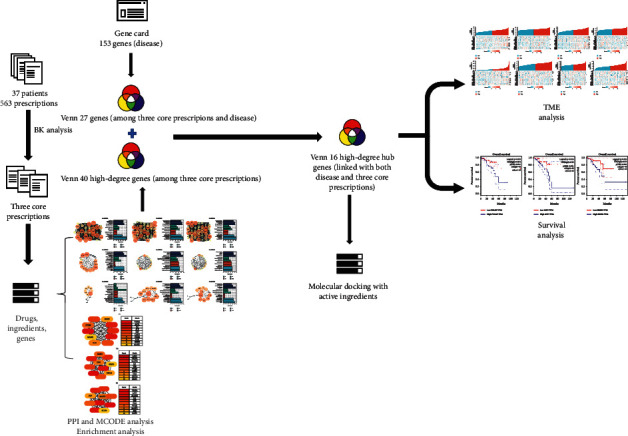
Study procedure.

The purpose of this study was to evaluate systematically and elucidate the composition of the TCM prescription applied at our hospital for the treatment of rectal cancer with Qi and blood deficiency “Zheng” (syndrome) using data mining to provide novel insights into the management and clinical treatment of this disease. Moreover, this study applied the methods of network pharmacology and molecular docking to explore the mechanisms of action for core prescriptions used to treat rectal cancer. The study procedure is shown in Figure 1.

## 2. Methods

### 2.1. Patient Information

The data used for this study were extracted from the medical records of outpatients and inpatients with rectal cancer in Ruijin Hospital affiliated with Shanghai Jiaotong University School of Medicine from January 2017 to February 2021. This study was a retrospective study. The protocols were approved by the Institutional Human Ethics Committee, Ruijin Hospital Affiliated with Shanghai Jiaotong University School of Medicine.

### 2.2. Study Enrollment Criteria

Patients with stage II, III, or IV rectal cancer with a clear pathological diagnosis were included in the study. There were no restrictions with regard to age or sex.

### 2.3. Exclusion Criteria

The following patients were excluded from the analysis: (1) patients who did not meet the inclusion criteria or who had incomplete data with regard to determinations of treatment efficacy; (2) pregnant or lactating women; (3) patients with a known allergy or those that presented with an adverse reaction during treatment; (4) patients diagnosed with psychiatric conditions; and (5) patients with severe comorbid conditions, such as cardiovascular disease, cerebrovascular disease, impaired liver or kidney function, or a hematopoietic system disease.

### 2.4. Data Processing and Implementing Algorithms

The following data were collected from the patient's original medical records to establish a database: general information (name, sex, age, and course of disease), clinical symptoms, laboratory tests, diagnosis, tongue condition, pulse condition, and prescription (drug names and dosage). All data were double-checked by two operators (Shiyu Ma and Lin Zheng) to ensure the accuracy, consistency, and completeness of the data entered. A network analysis toolbox of MATLAB was used to build a drug-dispensing network, and the Bron–Kerbosch (BK) algorithm was used to mine the core prescriptions for a minimum of eight drugs and a support threshold of 0.03.

### 2.5. Evaluation of Core Prescriptions

The confidence of the mined core prescriptions was evaluated using the confidence based on the whole network (CMBN) score and was supported by the *α* confidence level [15]. Both values ranged from 0 to 1, and higher values indicated higher confidence. The specific algorithm used was as follows:

(1) $$\begin{array}{c} \text{CMBN}=\sum_{i=1}^{n}\text{number }\left(\text{BF}\cap X_{i}\right)/\text{number }\left(\text{BF}\right)/n, \end{array}$$

where number is the counting function and the numerator indicates the number of drugs shared by the “core prescription” BF (where number is the counting function and the numerator represents the ratio of the number of drugs shared by the BF of prescription *X*_*i*_ to the total number of drugs in the BF). The larger CMBN value of each group is the final core prescription.

### 2.6. Disease-Associated Genes

In this work, rectal cancer was considered a disease, and associated genes were collected from the GeneCards database.

### 2.7. Ingredient Preparation from Core Prescriptions and Target Prediction

Information about the chemical ingredients of the three core prescriptions (designated P1, P2, and P3) was gathered from the following literature and data sources: STITCH database, Herb Ingredients' Targets (HIT) database, Chinese Academy of Sciences Chemistry database, Traditional Chinese Medicine Systems Pharmacology database, and the Traditional Chinese Medicine Integrated Database (TCMID).

We collected the chemical ingredients and their structures from the PubChem database (https://pubchem.ncbi.nlm.nih.gov/). The quantitative estimate of drug-likeness (QED) was calculated to prescreen pharmaceutically active compounds in the P1, P2, and P3 prescriptions. Those compounds with QEDs >0.3 were chosen.

Potential targets of these ingredients were investigated and analyzed using HERB (http://herb.ac.cn/). HERB [19] is a high-throughput experimental data and reference-guided database of TCM. It is the most comprehensive list of herbs and ingredients created to date and combines data from multiple TCM databases. To date, the database contains 17,886 TCM-related papers published from 2011 by PubMed text mining and manually associates 1,241 gene targets and 494 modern diseases for 473 herb/ingredient combinations extracted from 1,966 of the published references. Using data from HERB, we calculated the indirect associations for each herb, ingredient, and targets relationship using Fisher's exact test or statistical inference, adopted from SymMap. HERB provides TCM herb-/ingredient-related information based on manual curation of novel references published within the past decade, which bridges a large gap since the creation of the HIT (2011) and TCMID (2012) databases [19]. We collected targets identified using Fisher's exact test (false discovery rate (FDR) < 0.05) from prescriptions P1, P2, and P3 separately.

### 2.8. Molecular Docking

PSOVina (http://cbbio.cis.umac.mo) [20, 21] is a hybrid model that combines particle swarm optimization global search and Broyden–Fletcher–Goldfarb–Shanno local search methods in AutoDock Vina to address the conformational search problems in docking studies. It has the advantage of reducing execution time (by 51%–60%) without compromising the prediction accuracies in the docking and virtual screening experiments.

For each docking assay, six human receptors were retrieved from the Protein Data Bank (PDB; http://www.wwpdb.org/), including human interleukin (IL)-6 (PDB ID: 1alu), human MYC (PDB ID: 1nkp), human matrix metalloproteinase 9 (MMP9; PDB ID: 1l6j), human IL-10 (PDB ID: 1lk3), human ILIB (PDB ID: 4dep), human transforming growth factor (TGF)-B1 (PDB ID: 3kfd), and human PPARG (PDB ID: 3e00).

### 2.9. Enrichment Analysis and Network Construction

To investigate potential pathways regulated by P1, P2, and P3, enrichment analysis was carried out to identify the significant biological profiles of P1, P2, and P3. The hypergeometric *P* value has been widely used to investigate significant gene expression patterns based on predefined functional terms. In this study, we performed Gene Ontology (GO) enrichment analysis for molecular function (MF), cellular component (CC), and biological process (BP) analyses. Pathway enrichment analysis was based on the Kyoto Encyclopedia of Genes and Genomes (KEGG) pathway database, Disease Ontology (DO) enrichment analysis, and the Reactome database.

Analysis of the functional interactions between proteins may provide insights into the mechanisms of P1, P2, and P3 activity, and a protein-protein interaction (PPI) network was predicted using the Search Tool for the Retrieval of Interacting Genes (STRING; http://string-db.org; version 11.0) online database. A combined score >0.4 was considered statistically significant. The plug-in app Molecular Complex Detection (MCODE; version 1.4.2) of Cytoscape (version 3.7.0; https://cytoscape.org/) was applied for clustering a given network on the basis of its topology to identify densely connected regions. The PPI networks were drawn using Cytoscape, and the significant modules in the PPI networks were identified using MCODE. The criteria for selection were as follows: MCODE scores > 5, degree cutoff = 2, node score cutoff = 0.2, max depth = 100, and k-score = 2. Subsequently, KEGG and GO enrichment analyses were performed for genes in these modules. Hub genes were defined as nodes with degrees ≥10 [21].

### 2.10. Survival Analysis and Genes Act on Tumor Microenvironment Analysis

The relationship between the expression level of the hub genes and the prognosis of rectal cancer and the correlation of this relationship with different core genes in the tumor microenvironment were analyzed using The Cancer Genome Atlas rectal adenocarcinoma (READ) database (https://portal.gdc.cancer.gov/). GEPIA2 (http://gepia2.cancer-pku.cn/#index) was applied for the survival analysis. TCGA-READ (RNAseq data of level 3 HTSeq-FPKM) was used to study the correlation analysis between genes and other genes after log2 transformation. Results were interpreted with ggplot2 in the *R* package (version 3.6.3). *P* values < 0.05 (^*∗*^), 0.01 (^*∗∗*^), and 0.001 (^*∗∗∗*^) indicated different levels of significance in the relationship between genes and genes.

## 3. Results

### 3.1. Patient Characteristics and Demographics

A total of 563 prescription medication items were collected from 37 patients with rectal cancer who had deficiencies of both Qi and blood. The average number of medications per single prescription was 22 (±4). Most patients were elderly; the average age was 66.69 (±8.92) years. Twenty-three patients were men, and 14 were women. The 4 diagnostic conditions were distributed as follows: all 37 patients had a light red tongue and white color; 32 patients had a fine pulse at the left and right wrists; 1 patient had a sunken pulse at the left and right wrists; and 4 patients had a fine string pulse at the left and right wrists. Of these 37 patients, 11 had stage II disease; 12 had stage III; and 14 had stage IV disease. Six patients were receiving chemotherapy; 13 patients had disease progression and recurrence; and 18 patients were in a recovery period.

A total of 280 herbs were used in the 563 prescription medication preparations. The following were used most often: BaiZhu, HuangQi, BaiHuaSheSheCao, FuLing, ChenPi, YiYiRen (*coicis semen*), EZhu (*curcumae rhizoma*), LingZhi (*Ganoderma*), ShuYangQuan (*Solanum lyratum Thunb.*), GanCao, DangShen, and ShanZha (*crataegi fructus*). The top 20 herbs (frequency and degree) are listed in Supplementary S1.

### 3.2. Analysis of Core Prescriptions

**Figure 2:**
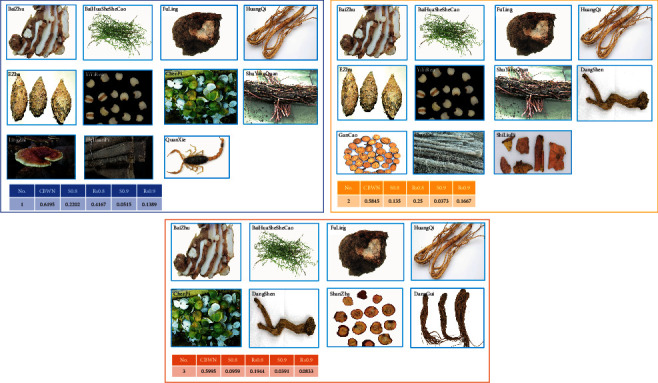
Three core prescriptions.

The BK algorithm was used to extract three core prescriptions (Supplement S2 and Figure 2). The herbs that appeared in each core prescription were BaiZhu, HuangQi, BaiHuaSheSheCao, and FuLing. This combination accounted for 36.4% of core prescription 1 (P1), 36.4% of core prescription 2 (P2), and 50% of core prescription 3 (P3). Herbs that appeared most often in the core prescriptions were EZhu and YiYiRen, which were the main drug components of “FuZhengXiaoJi” Fang, which we previously studied. In addition, 54.5% of herbs in P1 and P2 overlapped.

S0.8 and S0.9 represent the 80% and 90% proportions of patient prescriptions that overlapped with the core prescription. In P1, P2, and P3, the Rs0.8 was 0.4167, 0.25, and 0.194, respectively, indicating that 41.67%, 25%, and 19.4% of patients, respectively, used 80% of the herbs listed in P1, P2, and P3. We analyzed herbal prescriptions of patients in different subgroups (chemotherapy period, progression and recurrence period, and recovery period) and compared them with P1, P2, and P3. Among the six patients in the chemotherapy period group, four had prescriptions in which more than 80% of the herbs overlapped with those in P2, suggesting that P2 may be the core prescription for patients during chemotherapy. Among the 13 patients in the progression and recurrence period group, 6 had prescriptions in which more than 80% of the ingredients overlapped with P1, and 5 had prescriptions with 60%–80% overlap with P1, indicating that P1 may be the core prescription for patients in the progression and recurrence period. Among the 18 patients in the recovery period group, 6 had prescriptions in which more than 80% of the overlapped with P3, and 11 had prescriptions with 50%–80% overlap that indicates that P3 may be the core prescription for patients in the recovery period.

### 3.3. Identification of Chemical Composition in Three Core Prescriptions and Target Databases

After screening of the databases, removal of duplicate components, and integration of data, we confirmed 129, 191, and 164 active ingredients and 315, 439, and 405 major targets in P1, P2, and P3, respectively. Cytoscape 3.7.0 was applied to construct active ingredient-target network diagrams for the active ingredients and targets in P1, P2, and P3; Cytoscape identified the numbers of active ingredients with a degree rank value ≥ 200 for each prescription (*n* = 16 in P1, 30 in P2, and 21 in P3; Supplement S3).

**Figure 3:**
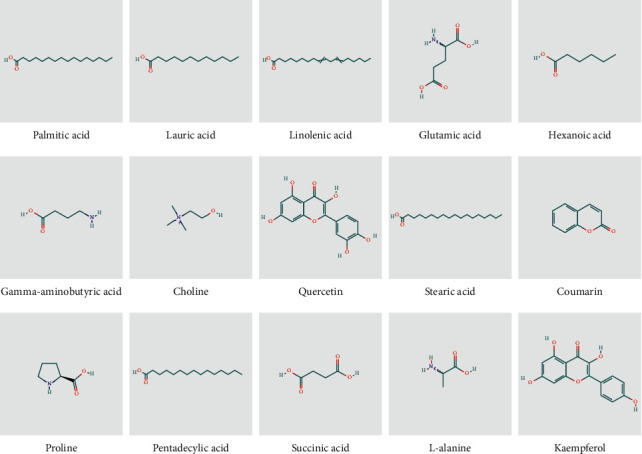
Fifteen ingredients found in three core prescriptions with degrees ≥200.

As shown in Figure 3 (and listed in Supplement S4), a total of 15 compounds had a common rank value ≥ 200 common to all three core prescriptions. These compounds were palmitic acid, lauric acid, succinic acid, glutamic acid, choline, linolenic acid, quercetin, proline, hexanoic acid, coumarin, L-alanine, stearic acid, gamma-aminobutyric acid, pentadecylic acid, and kaempferol. The components represented fatty acid compounds (saturated fatty acids, such as palmitic acid and lauric acid), unsaturated fatty acids (linolenic acid), and flavonoids (quercetin and kaempferol), which have anti-inflammatory, anti-oxidant, and anti-tumor effects. Amino acids included LPG, L-alanine, proline, gamma-aminobutyric acid, and glutamine. Therefore, it was inferred that the main active ingredients of core prescriptions involved fatty acids, amino acids, and flavonoids.

**Figure 4:**
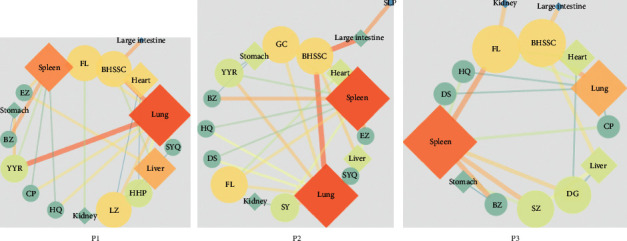
Herbal medicine: meridian network diagram. The symbol ○ indicates different herbal medicines and ◊ indicates different meridians.

Based on the Chinese Pharmacopoeia (2020 edition) and the Shanghai Concoction Specification (2018 edition), we constructed the Chinese herbal medicine network of core prescriptions using Cytoscape 3.7.0 (Figure 4). The size of each node in Figure 3 represents the size of the degree, which represents the number of nodes that directly interacted with each node in the network. The greater the degree of a node, the more data flows through the node; this distinction is the focus of core target screening. The color changes from cold to warm according to the value of betweenness centrality, which represents the shortest paths through a node. The higher the number, the warmer the color, indicating that the node has more data flowing through it and is an important node. The edge betweenness centrality is expressed by the thickness of the connection. The thicker the line, the shorter the path that contains the connection.

Figure 4 shows that the highest degrees associated with the three core prescriptions were the lung meridians, spleen meridians, and liver meridians. Most herbs belonged to these three meridians.

### 3.4. Enrichment Analysis of Core Prescription Target Interventions

**Figure 5:**
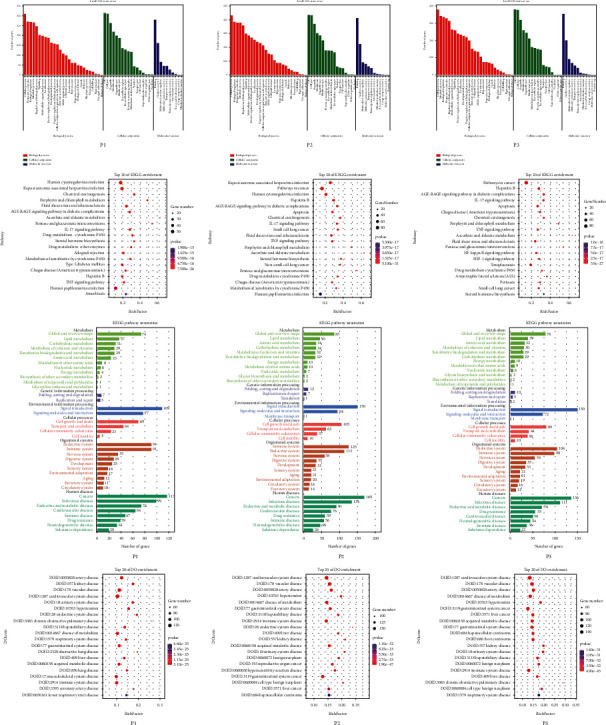
Gene Ontology, Kyoto Encyclopedia of Genes and Genomes, and Disease Ontology analyses of core prescriptions.

Targets of the P1, P2, and P3 prescriptions were subjected to GO enrichment analysis (Figure 5) by screening using a cutoff value FDR (adjusted *P* value) < 0.01 to identify the top 100 most significant ontology molecular functions (MFs) of the three core prescriptions, and Venn analysis was performed. We identified 72 biological functions of P1, 94 of P2, and 87 of P3 that overlapped with each other; these functions mainly involved proteins, enzymes, cytokine receptors, nuclear receptors, peptides, and cofactors. The main MF included binding to proteins, enzymes, cytokine receptors, nuclear receptors, peptides, and cofactors, which promotes activities involving protein dimerization, protein kinases, transcription factors, and neurotransmitter receptors. The enrichment of MF mainly focused on energy metabolisms, such as glucuronosyltransferase activity (GO:0015020), UDP-glycosyltransferase activity (GO:0008194), NADP binding (GO:0050661), and other MF pathways with effects on tumor immunity. Other MF pathways, such as TNF receptor superfamily binding (GO:0032813), TNF receptor binding (GO:0005164), and p53 binding (GO:0002039), also have a role in tumor immunity. P1 has effects on fatty acid-binding (GO:0005504), and P3 also promotes norepinephrine binding (GO:0051380) and growth factor receptor binding (GO:0070851).

Screening using an FDR (adjusted *P*) < 0.01 to identify the top 100 most significant biological processes (BPs) attributable to the three core prescriptions and subsequent Venn analysis revealed that 64 BPs of P1, 90 of P2, and 88 of P3 overlapped. The main BPs were as follows: (1) apoptosis-related effects of tumor, such as negative regulation of programmed cell death (GO:0043069), negative regulation of cell death (GO:0060548), regulation of cell death (GO:0010941), regulation of programmed cell death (GO:0043067), positive regulation of cell death (GO:0010942), and positive regulation of programmed cell death (GO:0043068); (2) oxidative stress, such as response to oxygen levels (GO:0070482) and cellular response to ROS (GO:0034614); (3) regulation of inflammatory response, inflammatory response (GO:0006954), chronic inflammatory response to antigenic stimulus (GO:0002439), and chronic inflammatory response (GO:0002544); (4) stimulation of immune function, such as regulation of immune system processes (GO:0002682), immune system processes (GO:0002376), and regulation of immune response (GO:0050776); and (5) angiogenesis-related BPs, such as VEGF-activated neuropilin signaling pathway (GO:0038190), VEGF-activated platelet-derived growth factor receptor signaling pathway (GO:0038086), and positive regulation of cell proliferation by the VEGF-activated platelet-derived growth factor receptor signaling pathway (GO:0038091).

The targets of the P1, P2, and P3 prescriptions were subjected to KEGG enrichment analysis (Figure 5), setting the threshold at an FDR (adjusted *P*) < 0.01. The KEGG enrichment analysis results were mainly associated with oncological diseases. The three core prescriptions converged on pathways such as the IL-17 signaling pathway (ko04657), the TNF signaling pathway (ko04668), and the VEGF signaling pathway (ko04370). P2 and P3 prescriptions also affected the apoptosis pathway (ko04210).

Setting the threshold at an FDR (adjusted *P*) _<_ 0.05, DO enrichment analysis (Figure 5) showed that the core prescription interventions were mainly related to tumors, blood circulation, and the immune system and included gastrointestinal system cancer (DOID:3119), gastrointestinal system disease (DOID:77), immune system disease (DOID:2914), cardiovascular system disease (DOID:1287), vascular disease (DOID:178), and artery disease (DOID:0050828).

Setting the threshold FDR (adjusted *P*) < 0.05, Reactome enrichment analysis (Supplement S5) showed that 7 of the top 20 core pathways in the core prescription, namely, glucuronidation (R-HSA-156588), extra-nuclear estrogen signaling (R-HSA-9009391), signaling by interleukins (R-HSA-449147), immune system (R-HSA-168256), IL-10 signaling (R-HSA-6783783), cytokine signaling in the immune system (R-HSA-1280215), and interleukin-4 and IL-13 signaling (R-HSA-6785807); all had significant effects.

In summary, using GO functional analysis, KEGG functional analysis, DO enrichment analysis, and Reactome enrichment analysis, our findings indicated that the core prescription targets mainly act on tumor apoptosis-related, immune anti-inflammatory effects, and oxidative stress effects. These functional and enrichment analysis results are consistent with activities involving the IL-17 signaling pathway, TNF signaling pathway, glucuronidation, IL-10 signaling, IL-4 and IL-13 signaling, and VEGF signaling, which may be the most relevant pathways activated by the P1, P2, and P3 prescriptions.

### 3.5. Analysis of the Target PPI Network for Core Prescriptions

The PPI networks of P1, P2, and P3 contained 291, 410, and 375 nodes, respectively, and 3,482, 6,629, and 5,828 edges, respectively. The average respective node degrees were 23.9, 32.3, and 31.1; the average respective local clustering coefficients were 0.501, 0.497, and 0.51; and the respective expected numbers of edges were 1,402, 2,805, and 2,468. Data were based on the PPI network enrichment threshold *P* < 1.0 × 10^−16^.

**Figure 6:**
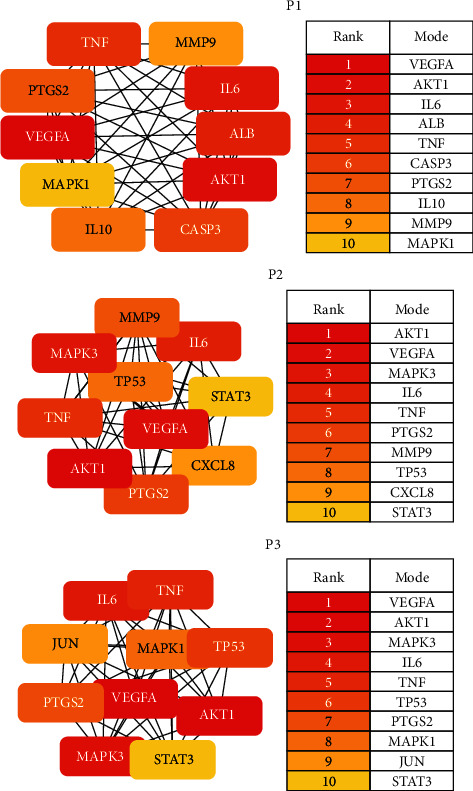
Hub genes in three core prescriptions.

**Figure 7:**
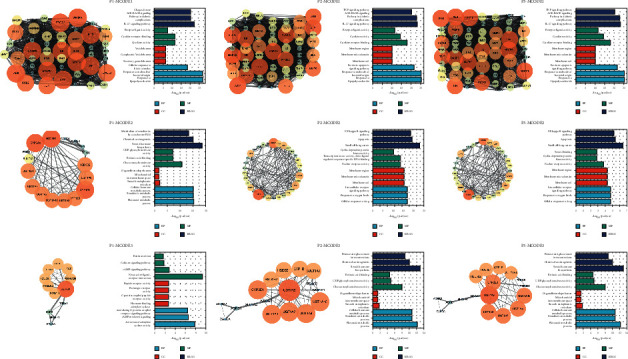
Molecular Complex Detection, Gene Ontology, and Kyoto Encyclopedia of Genes and Genomes analyses of P1, P2, and P3.

The results of functional analyses and MCODE and hub genes analyses are shown in Figures 6 and 7_._ As seen in Figure 6, the hub genes in the three core prescriptions were mainly AKT1, VEGFA, IL6, PTGS2, and TNF. In the MCODE analysis, the three most important modules of the three core prescriptions were selected for analysis. As seen in Figure 7, most of the genes in the three core prescriptions in module 1 enriched the IL-17 and TNF signaling pathways. Genes of P2 and P3 in module 2 were enriched in the NF-kB signaling pathway and apoptosis.

With regard to the core prescription screening, degrees with ≥50 nodes included 44 targets in P1, 92 targets in P2, and 77 targets in P3; of these, 40 targets were present in all three core prescriptions. The common targets included ACE, MMP2, TLR4, FOS, IFNG, AKT1, ALB, PPARG, IL10, SERPINE1, MAPK1, GPT, RELA, VCAM1, TP53, STAT1, HMOX1, NOS3, IL6, PTGS2, TNF, VEGFA, CXCL8, TGFB1, IL1B, ICAM1, REN, MYC, SOD1, JUN, BCL2L1, CASP3, CAT, MPO, MMP9, TIMP1, POMC, GCG, ESR1, and ERBB2.

**Figure 8:**
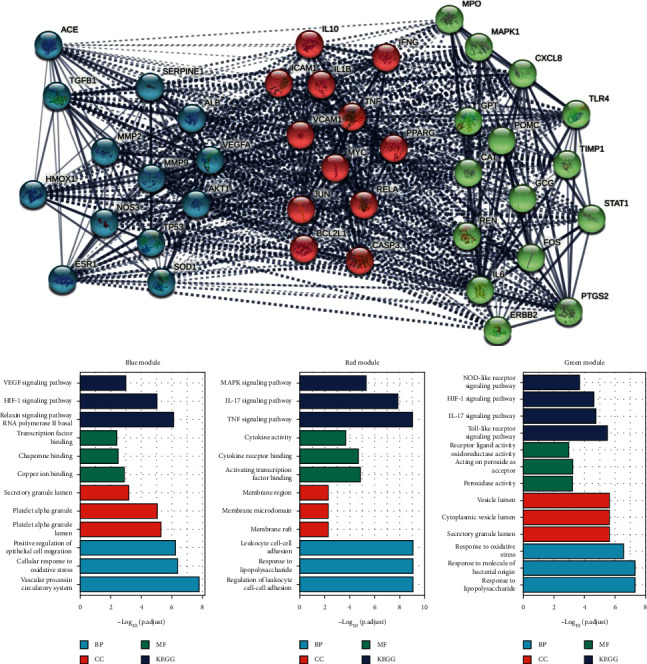
Protein-protein interaction analysis of 40 high-degree genes among the 3 core prescriptions and analysis of Gene Ontology and Kyoto Encyclopedia of Genes and Genomes in different high-degree gene modules.

The network topology analyses of targets with the highest node counts were evaluated using STRING by k-means clustering, and genes were classified into three groups (Figure 8). The three groups of KEGG analysis were also studied (Figure 8 and Supplementary S6). The targets in the green group were mainly related to autophagy and immune anti-inflammation responses (FOS, IL6, CXCL8, MAPK1, STAT1, TLR4, PTGS2, ERBB2, and TIMP1), whereas the targets in the blue cluster were mostly related to angiogenesis and migration (VEGFA, NOS3, and ATK1). The targets in the red target cluster were mostly involved in tumor apoptosis and immune activity. Of these identified genes, AKT1 plays a regulatory role in cell proliferation, differentiation, and metabolic functions; TNF is involved in lipid metabolism, cell proliferation, differentiation, apoptosis, and coagulation and plays an important role in various diseases, including autoimmune diseases, insulin resistance, and cancer. Activation of the MAPK cascade is central to a variety of signaling pathways and involves an important class of molecules that receives signals that are converted and transmitted by membrane receptors and carried into the nucleus to play a key role in many cell proliferation-related signaling pathways. The MAPK pathway is activated sequentially by three kinase enzymes that together regulate cell growth, differentiation, adaptation to environmental stress, inflammatory responses, and many other important processes.

### 3.6. Core Prescription for Rectal Cancer

**Figure 9:**
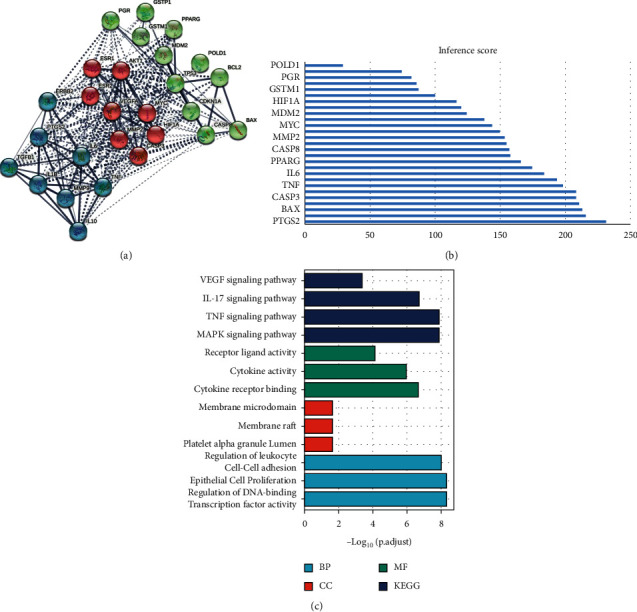
Protein-protein interaction (PPI) network of 27 coacting 27 genes: (a) PPI network of 27 coacting genes, (b) inference score of the common genes, and (c) Gene Ontology and Kyoto Encyclopedia of Genes and Genomes enrichment analysis of common genes.

A total of 134 targets were obtained by screening disease targets with relevance scores >50 in GeneCards. The PPI networks of P1, P2, and P3 were constructed by STRING, and the PPI networks of rectal cancer were merged and analyzed separately. The intersection of the three was taken to find the targets with their common effects, as shown in Figure 9 and Supplement S7.

The Venn analysis (Supplement S7) showed that PTGS2, PGR, BAX, CASP8, TGFB1, CASP3, BCL2, GSTP1, GSTM1, IL1B, MMP2, CDKN1A, POLD1, TP53, IL6, TNF, and ESR1, existed for all three core prescriptions and CRC. By PPI analysis and a k-means clustering algorithm, three main classes of targets were identified.

An analysis of biological pathways involving the 27 highest node targets (FDR < 0.01) was performed using the Reactome tool. Five main pathways were identified: cytokine signaling in the immune system (HSA-1280215), apoptosis (HSA-109581), hemostasis (HSA-109582), metabolism of angiotensinogen to angiotensin (HSA-2022377), and IL-6 signaling (HSA-1059683). In the KEGG analysis (FDR < 0.01), most of these pathways were related to tumor immunity and included the TNF, IL-17, and NF-kB signaling pathways, apoptosis, MAPK signaling, T-cell receptor signaling, platinum drug resistance, and resistance to epidermal growth factor receptor (EGFR) tyrosine kinase inhibitors.

The first group (indicated in green in Figure 9) involved 11 genes. Of these, CASP8, BCL2, BAX, and TP53 were all related to the intrinsic pathway for apoptosis and were also associated with the programmed cell death pathways. Reactome aggregation analysis (FDR < 0.01) found that the green target cluster was mainly associated with two pathways: transcriptional activation of cell cycle inhibitor p21 and transcriptional activation of p53-responsive genes. The major roles were attributed to CDKN1A and TP53 targets.

The second group (consisting of red targets in Figure 9) comprised eight genes. Reactome aggregation analysis (FDR < 0.01) showed that the red target group was mainly associated with four pathways: IL-4 and IL-13 signaling, ESR-mediated signaling, PI3K/AKT signaling in cancer, and signaling by VEGF. The main interventions involved immunity, hormone receptors, and angiogenesis.

The last category of blue targets was represented by TNF, IL6, IL10, IL1B, MMP9, TGFB1, PTGS2, and ERBB2. Reactome aggregation analysis (FDR < 0.01) found that the targets were mainly related to IL-related receptors, IL-10 signaling (R-HSA-6783783), signaling by ILs (R-HSA-449147), IL-4 and IL-13 signaling (R-HSA-6785807), and cytokine signaling in the immune system (R-HSA-1280215).

The CTD database was used to verify the relevance of the core prescription targets to rectal cancer, and an inference score was used to determine the degree of relevance. Twenty-six targets (96.3%) had scores >50, indicating that they were extremely relevant to the diseases and could be regarded as intervention targets in the core prescriptions for rectal cancer (Figure 9).

Additional analysis of the 27 targets that coacted against the disease and the 40 high-degree targets that coacted with 3 core prescriptions revealed that 16 of them, namely, MMP2, AKT1, PPARG, IL10, TP53, IL6, PTGS2, TNF, VEGFA, TGFB1, IL1B, MYC, CASP3, MMP9, ESR1, and ERBB2, were coacting targets (among the disease and 3 core prescriptions). The rank values of these 16 targets in each prescription and the inference score in the disease are shown in Supplement S8. Additional GO and KEGG enrichment analyses of these 16 targets showed that the most significant pathways were the MAPK, IL-17, TNF, and VEGF pathways (Figure 9).

### 3.7. Survival and Tumor Microenvironment Analysis

**Figure 10:**
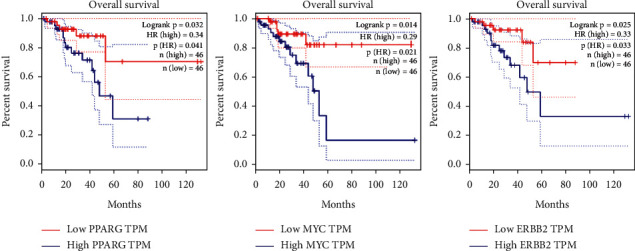
Overall survival associated with 16 genes.

**Figure 11:**
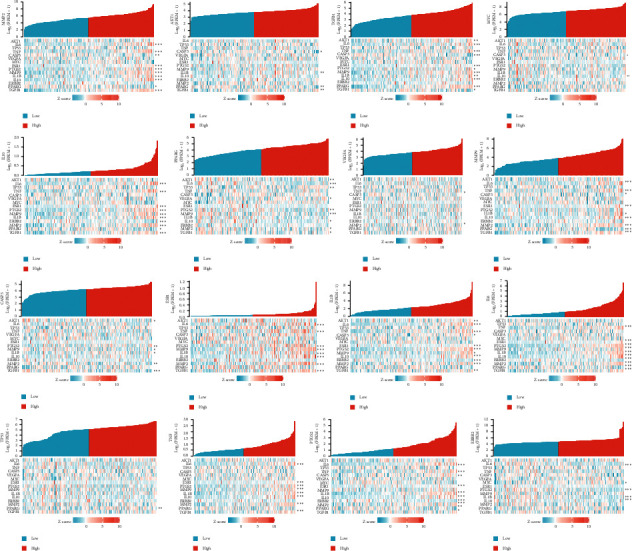
Tumor microenvironment in relation to 16 genes.

From the 16 genes, 3 genes (ERBB2, MYC, and PPARG) were linked with the survival of rectal cancer (Figure 10). TGFB1, IL1B, IL10, IL6, PTGS2, and PPARG closely interacted with the tumor microenvironment (Figure 11). In particular, TGFB1 and IL1B1 were related to 11 other genes in the microenvironment.

### 3.8. Molecular Docking Results

**Figure 12:**
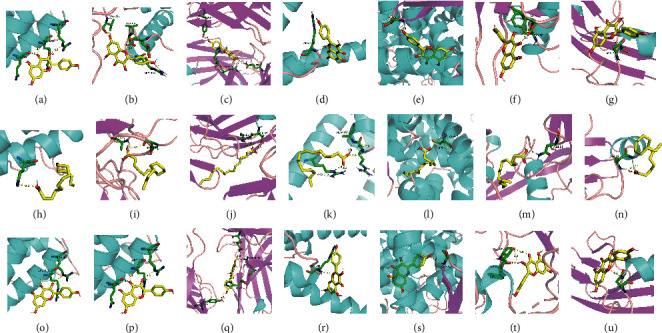
Molecular docking results: (a–g) lauric acid and interleukin (IL)-6, MMP9, IL-10, MYC, PPARG, TGFB1, and ILIB, respectively; (h–n) quercetin and IL-6, MMP9, IL-10, MYC, PPARG, TGFB1, and ILIB, respectively; and (o–u) kaempferol and IL-6, MMP9, IL-10, MYC, PPARG, TGFB1, and ILIB, respectively.

The docking results of the main 15 active ingredients in the core prescription with genes are shown in Figure 12 and Supplement S9. The smaller the binding energy value, the more stable the structure after binding. Among the ingredients, quercetin, kaempferol, and lauric acid had good binding energy to each target, suggesting that these three compounds play a key role in the treatment of rectal cancer.

## 4. Discussion

In this study, we identified the 3 core prescriptions (P1, P2, and P3) used to treat rectal cancer. We also found that P1, P2, and P3 may be potential core prescriptions for patients during the tumor progression and recurrence periods, the chemotherapy period, and the recovery period, respectively. The results revealed that the core prescriptions are involved in three major biological processes, tumor apoptosis, angiogenesis, and immune anti-inflammatory effects, through 15 main active ingredients and 16 potential targets and that the prescriptions activated the following pathways: signaling by ILs [22], MAPK signaling, and VEGF signaling.

### 4.1. TCM Perspective of Three Core Prescriptions

A deficiency of cellular immune function in patients with malignant tumors is closely associated with the onset, development, and prognosis of tumors [23]. In TCM, the main mechanism of rectal cancer is a Qi and blood deficiency combined with a spleen and stomach deficiency. Qi and blood are defined as the cornerstones of the fight against disease (i.e., good spleen and stomach function helps improve Qi and blood deficiencies) [24]. From the perspective of TCM, three core prescriptions focus on the lung, spleen, and liver meridians. The herbal components in the three core prescriptions are associated with the FuZhengXiaoJi prescription and include HuangQi, DangShen, BaiHuaSheSheCao, EZhu, LingZhi, YiYiRen, GanCao, BaiZhu, FuLing, ChenPi, and QuanXie (scorpio). These herbs have been used to treat rectal cancers for many years in our Chinese Medicine Department [25–27]. HuangQi improves Qi and lung deficiencies. BaiHuaSheSheCao and EZhu act on stasis, detoxify, and relieve pain. LingZhi and FuLing improve the immune system. FuZhengXiaoJi treatment can alleviate adverse reactions subsequent to chemotherapy treatment. Furthermore, the overall survival of patients with rectal cancer can be prolonged using FuZhengXiaoJi therapy at different tumor stages. The rate of recurrence and metastasis in stages II and III can be reduced, while progression-free survival in patients with stage IV disease can be extended; overall, survival can also be prolonged by FuZhengXiaoJi after surgical intervention and chemotherapy [28].

We analyzed herbal prescriptions for patients in different subgroups (chemotherapy period, progression and recurrence period, and recovery period), and compared them with P1, P2, and P3. P1 may be the core prescription for patients during the progression and recurrence period. Patients in the progression and recurrence period may be anxious, be irritable, and have insomnia because of the disease progression and recurrence, and HeHuanPi (*albiziae cortex*) can be used for these patients to improve sleep and anxiety symptoms. In addition, QuanXie, a component of P1 can be used to prevent cancer metastasis and improve the poor prognosis [29]. P2 may be the core prescription for patients during chemotherapy. The main drugs used by patients during chemotherapy are oxaliplatin and capecitabine, and oxaliplatin commonly causes diarrhea. ShiLiuPi (*granati pericarpium*) stops diarrhea and bleeding, so it may improve the adverse effect of oxaliplatin. P3 may be the core prescription for patients during the recovery period. The use of Qi-tonifying and spleen-invigorating herbs, such as DangShen, ShanZha, and ChenPi, in P3, helps improve the gastrointestinal function of convalescent patients and promotes recovery.

### 4.2. Potential Mechanism of Core Prescriptions

The main function of the three core prescriptions is to improve immune system function and defer tumor cell migration and invasion. Three potential IL family (IL-6, IL-10, and IL-1B) genes were common to the prescriptions. IL is an important immune and anti-inflammatory cytokine controlling cell growth, differentiation, and migration. IL-6 is a key proinflammatory cytokine within the gut [30], and it exerts a marked effect on the tumor microenvironment in a wide range of cancers [31]. It has been reported to play an important role in immune regulation, the inflammatory response, and the epithelial-mesenchymal transition (EMT) of tumor cells, processes that have been associated with inflammatory bowel disease as well as colon and rectal cancers [32]. Notably, tumor-associated macrophage-derived IL-6 binds to receptor/glycoprotein 130 (gp130) and upregulates Janus kinase (JAK)-STAT3 signaling in CRC cells, leading to increased EMT and chemoresistance, which promotes the proliferation and invasion of CRC cells [33].

IL-10 functions as an essential immunoregulator in inflammation and inflammation-associated cancer. IL-10 mainly blocks NF*κ*B activity in intestinal epithelial cells through the JAK-STAT signaling pathway. It is well accepted that NF*κ*B plays a central role in inflammatory and innate immune responses, and the inhibition of NF*κ*B activity may interfere with carcinogenesis and slows tumor development.

We identified AKT1, MMP2, and MMP9 as potential targets. Some studies have found that Akt1 signaling significantly suppresses the expression of MMP2, MMP9, HIF1*α*, and VEGF in colorectal carcinoma cells. Our data suggest a significant role for Akt1 in tumor cell migration and invasion [34]. Akt1 participates in regulating the VEGF signaling pathway to promote tumor angiogenesis, development, and metastasis; in particular, the inhibition of Akt1 reduces the secretion of VEGF.

### 4.3. Active Ingredients with Potential Targets

The molecular docking results found that quercetin, apigenin, and other flavonoids exhibit good binding energy for each target, indicating that they have potential as drug treatments, though pharmacodynamic studies are needed. Flavonoids could affect cancer risk through anti-inflammatory and anti-tumor activities, mainly by inhibiting cycloxygenase-2 (COX2) in colon cancer cells, which is associated with a reduced risk of CRC [35]. Flavonoids induce apoptosis and suppress the growth of colon cancer cells by inhibiting the COX2- and Wnt/EGFR/NF*κ*B-signaling pathways, which play crucial roles in CRC [36]. Quercetin inhibits tyrosine kinase activity, thus downregulating cell proliferation. It has been suggested that the free radical scavenging properties of flavonoids are closely related to beneficial effects on cancer risk [37, 38]. In addition, our findings indicated that fatty acids may have potential anti-CRC activity [39]. Lauric acid displayed preferential antineoplastic properties, including induction of apoptosis, in a CRC cell line [40]. Lauric acid can improve the sensitization of cetuximab in KRAS/BRAF-mutated CRC cells by inducing microRNA-378 expression, and it has no adverse effects on the cardiovascular system [40].

### 4.4. Core Genes Linked with Survival and Tumor Microenvironment

From the 16 core genes identified in this study, we found 3 genes linked with the survival of rectal cancer and 5 genes related to the tumor microenvironment [41]. Some studies have noted that the MYC/MNX1-AS1/YB1 signaling pathway can drive proliferation and affect survival in colorectal cancer [42]. We found that TGF-*β*1 had a significant impact on multiple genes in the tumor microenvironment. In patients with CRC, a stroma-expressed gene program enriched for TGF-*β* has been linked to poor prognosis and metastasis formation. Thus, TGF-*β* signaling plays a key role in instructing the tumor microenvironment in late-stage CRC. TGF-*β*, TNF*α*, and NF*κ*B are capable of cooperatively mediating the development of EMT. The integrated pathway involving TGF-*β*/Snail with TNF*α*/NF*κ*B may be the principal axis that associates cancer cells to their microenvironment during the EMT process and exerts a critical role in CRC development and prognosis, which results in a poor prognosis for patients [43]. TGF-*β*1 and IL-6 are crucial cytokines for Th17 differentiation and are upregulated in the tumor tissue of patients with CRC. CRC-conditioned macrophages regulate the EMT program to enhance CRC cell migration and invasion by secreting IL-6 [44].

We also evaluated the impact of ATK1 and VEGFA activities on the tumor microenvironment. Angiogenesis is highly regulated by various factors involved in different signaling pathways. Among these pathways, the VEGF and the TGF-*β* families of proteins are especially relevant. The AKT/HIF1*α* signaling pathway, which regulates the expression of VEGF to promote angiogenesis, also provides potential therapeutic opportunities for the treatment of CRC [45]. PPAR*γ* agonists can regulate glucose and lipid metabolism. Current evidence indicates that obesity and overweight status are risks that contribute to CRC. PPAR*γ* can participate in primordial cell activation and development, possibly mediated in part by the PI3K/AKT signaling pathway. PPARG gene activity might be one of the targets of miRNA-34a and a conceivable therapeutic target for CRC [46].

## 5. Conclusion

Our results support and enhance the current understanding of the therapeutic effects of TCM “Zheng” (syndrome) on rectal cancer. We have demonstrated an effective strategy combining network pharmacology and data mining to understand the mechanisms of action of core prescriptions for patients with rectal cancer and Qi and blood deficiency syndrome. Our findings may facilitate the generation of new hypotheses to reveal the mechanisms of TCM treatment for rectal cancer.

## Data Availability

The datasets used for the current study are available from the corresponding author upon request.

## Supplementary Materials

Supplementary Materials S1: top 20 herbs in three core prescriptions; S2: three core prescriptions; S3: core compounds with a common rank value > 200 in the three core prescriptions; S4: most important active ingredients in core prescription relevant to the target; S5: Venn map of the top 20 Reactome pathways in the core prescription; S6: forty high-degree targets from enrichment analysis based on the Kyoto Encyclopedia of Genes and Genomes pathway; S7: coacting genes in three core prescriptions; S8: sixteen high-degree hub genes linked with both rectal cancer and three core prescriptions; and S9: molecular docking results of active ingredients in core prescriptions.
