# Supplementary material for: Data Mining, Network Pharmacology, and Molecular Docking Explore the Effects of Core Traditional Chinese Medicine Prescriptions in Patients with Rectal Cancer and Qi and Blood Deficiency Syndrome
Source: Evid Based Complement Alternat Med. 2021 Aug 2;2021:1353674. doi: 10.1155/2021/1353674 (PMC8360715; doi:10.1155/2021/1353674)
Supplement: Supplementary Materials — S1: top 20 herbs in three core prescriptions; S2: three core prescriptions; S3: core compounds with a common rank value > 200 in the three core prescriptions; S4: most important active ingredients in core prescription relevant to the target; S5: Venn map of the top 20 Reactome pathways in the core prescription; S6: forty high-degree targets from enrichment analysis based on the Kyoto Encyclopedia of Genes and Genomes pathway; S7: coacting genes in three core prescriptions; S8: sixteen high-degree hub genes linked with both rectal cancer and three core prescriptions; and S9: molecular docking results of active ingredients in core prescriptions. [file 1353674.f1.zip › 1353674.f1/S1 Top 20 herbs in three core prescriptions.docx]

S1 Top 20 herbs in three core prescriptions

| No. | Herb | Degree | frequency | No. | Herb | Degree | frequency |
| --- | --- | --- | --- | --- | --- | --- | --- |
| 1 | BaiZhu(*ATRACTYLODIS MACROCEPHALAE*  *RHIZOMA*) | 286 | 551 | 11 | ShuYangQuan(*Solanum lyratum Thunb.*) | 200 | 316 |
| 2 | HuangQi(*ASTRAGALI RADIX*) | 278 | 526 | 12 | LiuShenQu | 190 | 184 |
| 3 | BaiHuaSheSheCao(*Hedyotis diffusa Willd*) | 265 | 503 | 13 | GanCao(*GLYCYRRHIZAE RADIX ET RHIZOMA*) | 190 | 303 |
| 4 | FuLing(*PORIA*) | 253 | 424 | 14 | DangShen(*CODONOPSIS RADIX*) | 188 | 186 |
| 5 | ChenPi(*CITRI RETICULATAE PERICARPIUM*) | 232 | 307 | 15 | GeGen(*PUERARIAE LOBATAE RADIX*) | 181 | 117 |
| 6 | YiYiRen(*COICIS SEMEN*) | 226 | 397 | 16 | DaoYa(*ORYZAE FRUCTUS GERMINATUS*) | 179 | 226 |
| 7 | BanZhiLian(SCUTELLARIAE BARBATAE HERBA) | 214 | 288 | 17 | XianHeCao(*AGRIMONIAE HERBA*) | 177 | 105 |
| 8 | EZhu(*CURCUMAE RHIZOMA*) | 210 | 230 | 18 | ShanZha(*CRATAEGI FRUCTUS*) | 175 | 155 |
| 9 | LingZhi(*GANODERMA*) | 209 | 173 | 19 | ShiHu(*DENDROBII CAULIS*) | 169 | 149 |
| 10 | HuangQin(*SCUTELLARIAE RADIX*) | 203 | 292 | 20 | BaiShao(*PAEONIAE RADIX ALBA*) | 168 | 124 |
