# Supplementary material for: Data Mining, Network Pharmacology, and Molecular Docking Explore the Effects of Core Traditional Chinese Medicine Prescriptions in Patients with Rectal Cancer and Qi and Blood Deficiency Syndrome
Source: Evid Based Complement Alternat Med. 2021 Aug 2;2021:1353674. doi: 10.1155/2021/1353674 (PMC8360715; doi:10.1155/2021/1353674)
Supplement: Supplementary Materials — S1: top 20 herbs in three core prescriptions; S2: three core prescriptions; S3: core compounds with a common rank value > 200 in the three core prescriptions; S4: most important active ingredients in core prescription relevant to the target; S5: Venn map of the top 20 Reactome pathways in the core prescription; S6: forty high-degree targets from enrichment analysis based on the Kyoto Encyclopedia of Genes and Genomes pathway; S7: coacting genes in three core prescriptions; S8: sixteen high-degree hub genes linked with both rectal cancer and three core prescriptions; and S9: molecular docking results of active ingredients in core prescriptions. [file 1353674.f1.zip › 1353674.f1/S2 Three Core prescriptions.docx]

S2 Three Core prescriptions

| No. | CBWN | S0.8 | Rs0.8 | S0.9 | Rs0.9 | component |
| --- | --- | --- | --- | --- | --- | --- |
| 1 | 0.6195 | 0.2202 | 0.4167 | 0.0515 | 0.1389 | BaiZhu (*Atractylodis macrocephalae Rhizoma*),  EZhu(*CURCUMAE RHIZOMA*),  YiYiRen(*COICIS SEMEN*),  ChenPi*(Citri reticulatae Pericarpium*),  BaiHuaSheSheCao (*Hedyotis diffusa Willd*),  ShuYangQuan(*Solanum lyratum Thunb.*),  FuLing (*Poria*),  LingZhi(*GANODERMA*),  HuangQi(*Astragali radix*),  HeHuanPi(*ALBIZIAE CORTEX*),  QuanXie(*SCORPIO*) |
| 2 | 0.5845 | 0.135 | 0.25 | 0.0373 | 0.1667 | BaiZhu (*Atractylodis macrocephalae Rhizoma*),  EZhu(*CURCUMAE RHIZOMA*),  YiYiRen(*COICIS SEMEN*),  BaiHuaSheSheCao (*Hedyotis diffusa Willd*),  ShuYangQuan(*Solanum lyratum Thunb.*),  FuLing (*Poria*),  GanCao(*GLYCYRRHIZAE RADIX ET RHIZOMA*), HuangQi(*Astragali radix*),  DangShen(*CODONOPSIS RADIX*),  ShanYao(*DIOSCOREAE RHIZOMA*),  ShiLiuPi(*GRANATI PERICARPIUM*) |
| 3 | 0.5995 | 0.0959 | 0.1944 | 0.0391 | 0.0833 | BaiZhu(*Atractylodis macrocephalae Rhizoma*),  ChenPi(*Citri reticulatae Pericarpium*),  BaiHuaSheSheCao (*Hedyotis diffusa Willd*),  FuLing (*Poria*),  HuangQi*(Astragali radix*),  DangShen(*CODONOPSIS RADIX*),  ShanZha(*CRATAEGI FRUCTUS*),  DangGui(*ANGELICAE SINENSIS RADIX*) |
