# Supplementary material for: Data Mining, Network Pharmacology, and Molecular Docking Explore the Effects of Core Traditional Chinese Medicine Prescriptions in Patients with Rectal Cancer and Qi and Blood Deficiency Syndrome
Source: Evid Based Complement Alternat Med. 2021 Aug 2;2021:1353674. doi: 10.1155/2021/1353674 (PMC8360715; doi:10.1155/2021/1353674)
Supplement: Supplementary Materials — S1: top 20 herbs in three core prescriptions; S2: three core prescriptions; S3: core compounds with a common rank value > 200 in the three core prescriptions; S4: most important active ingredients in core prescription relevant to the target; S5: Venn map of the top 20 Reactome pathways in the core prescription; S6: forty high-degree targets from enrichment analysis based on the Kyoto Encyclopedia of Genes and Genomes pathway; S7: coacting genes in three core prescriptions; S8: sixteen high-degree hub genes linked with both rectal cancer and three core prescriptions; and S9: molecular docking results of active ingredients in core prescriptions. [file 1353674.f1.zip › 1353674.f1/S4 The most important active ingredients in core prescription that are relevant to the target.docx]

S4 The most important active ingredients in core prescription that are relevant to the target

| PubChem CID | ingredients | Degree(P1) | Degree(P2) | Degree(P3) |
| --- | --- | --- | --- | --- |
| 985 | palmitic acid | 4118 | 5100 | 6564 |
| 3893 | lauric acid | 1448 | 780 | 1431 |
| 3931 | linolenic acid | 931 | 918 | 596 |
| 33032 | Glutamic acid | 616 | 1484 | 662 |
| 8892 | hexanoic acid | 556 | 556 | 423 |
| 119 | gamma-aminobutyric acid | 514 | 687 | 287 |
| 305 | choline | 466 | 599 | 662 |
| 5280343 | quercetin | 465 | 1206 | 556 |
| 5281 | stearic acid | 366 | 301 | 306 |
| 323 | coumarin | 366 | 462 | 380 |
| 145742 | Proline | 340 | 708 | 448 |
| 13849 | PENTADECYLIC ACID | 270 | 270 | 270 |
| 1110 | succinic acid | 249 | 334 | 697 |
| 5950 | LPG, L-Alanine | 238 | 800 | 318 |
| 5280863 | kaempferol | 207 | 600 | 254 |
