# Supplementary material for: Data Mining, Network Pharmacology, and Molecular Docking Explore the Effects of Core Traditional Chinese Medicine Prescriptions in Patients with Rectal Cancer and Qi and Blood Deficiency Syndrome
Source: Evid Based Complement Alternat Med. 2021 Aug 2;2021:1353674. doi: 10.1155/2021/1353674 (PMC8360715; doi:10.1155/2021/1353674)
Supplement: Supplementary Materials — S1: top 20 herbs in three core prescriptions; S2: three core prescriptions; S3: core compounds with a common rank value > 200 in the three core prescriptions; S4: most important active ingredients in core prescription relevant to the target; S5: Venn map of the top 20 Reactome pathways in the core prescription; S6: forty high-degree targets from enrichment analysis based on the Kyoto Encyclopedia of Genes and Genomes pathway; S7: coacting genes in three core prescriptions; S8: sixteen high-degree hub genes linked with both rectal cancer and three core prescriptions; and S9: molecular docking results of active ingredients in core prescriptions. [file 1353674.f1.zip › 1353674.f1/S6 Forty High Degree targets enrichment analysis based on KEGG pathway.docx]

S6 Forty High Degree targets enrichment analysis based on KEGG pathway

| ID | descriptions | count | FDR |
| --- | --- | --- | --- |
| hsa04933 | AGE-RAGE signaling pathway in diabetic complications | 17 | 5.11*10^-26^ |
| hsa04668 | TNF signaling pathway | 13 | 2.79*10^-18^ |
| hsa04657 | IL-17 signaling pathway | 12 | 2.97*10^-17^ |
| hsa04066 | HIF-1 signaling pathway | 12 | 4.94*10^-17^ |
| hsa04620 | Toll-like receptor signaling pathway | 11 | 3.42*10^-15^ |
| hsa04380 | Osteoclast differentiation | 11 | 2.04*10^-14^ |
| hsa04010 | MAPK signaling pathway | 13 | 1.82*10^-13^ |
| hsa04926 | Relaxin signaling pathway | 10 | 1.30*10^-12^ |
| hsa04064 | NF-kappa B signaling pathway | 9 | 3.19*10^-12^ |
| hsa01522 | Endocrine resistance | 9 | 3.69*10^-12^ |
| hsa04659 | Th17 cell differentiation | 9 | 6.49*10^-12^ |
| hsa04621 | NOD-like receptor signaling pathway | 10 | 1.09*10^-11^ |
| hsa04210 | Apoptosis | 9 | 6.15*10^-11^ |
| hsa04932 | Non-alcoholic fatty liver disease (NAFLD) | 9 | 1.34*10^-10^ |
| hsa04218 | Cellular senescence | 9 | 1.83*10^-10^ |
| hsa04660 | T cell receptor signaling pathway | 8 | 1.93*10^-10^ |
| hsa04151 | PI3K-Akt signaling pathway | 11 | 4.38*10^-10^ |
| hsa01524 | Platinum drug resistance | 6 | 3.61*10^-08^ |
| hsa01521 | EGFR tyrosine kinase inhibitor resistance | 6 | 6.27*10^-08^ |
| hsa04658 | Th1 and Th2 cell differentiation | 6 | 1.21*10^-07^ |
