# Supplementary material for: Data Mining, Network Pharmacology, and Molecular Docking Explore the Effects of Core Traditional Chinese Medicine Prescriptions in Patients with Rectal Cancer and Qi and Blood Deficiency Syndrome
Source: Evid Based Complement Alternat Med. 2021 Aug 2;2021:1353674. doi: 10.1155/2021/1353674 (PMC8360715; doi:10.1155/2021/1353674)

**S7 Co-acting genes in three core prescriptions.**

|  | **Co-acting genes** |
| --- | --- |
| P1,P2,P3,cancer | PTGS2,PGR,BAX,CASP8,TGFB1,CASP3,BCL2,GSTP1,GSTM1,IL1B,MMP2,CDKN1A,POLD1,TP53,IL6,TNF,ESR1,ESR2,PPARG,IL10,HIF1A,MMP9,MYC,VEGFA,AKT1,ERBB2,MDM2 |
| Rectalcancer,P3,P2 | STAT3,EGFR,CCND1,CDK4,RB1,BIRC5,ATM,IGF1,ABCB1,SRC,CHEK2 |
| Rectalcancer,P3 | MSH3 |
| Rectalcancer,P1 | CDH1 |
| Rectalcancer,P2 | AR,CYP1A1 |


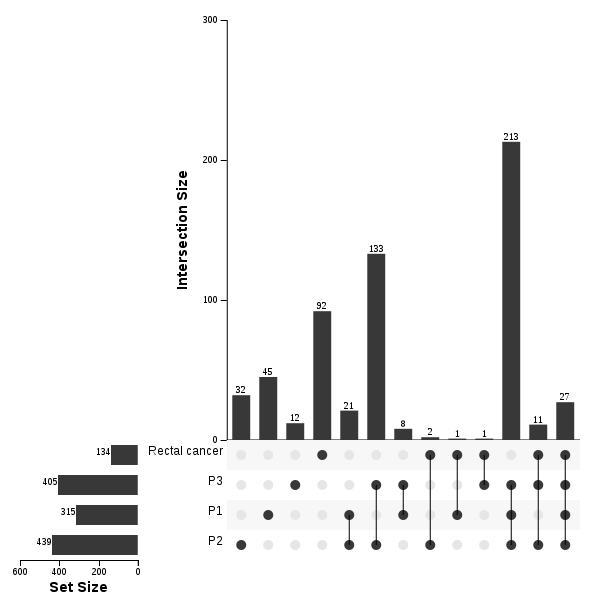

Supplement: Supplementary Materials — S1: top 20 herbs in three core prescriptions; S2: three core prescriptions; S3: core compounds with a common rank value > 200 in the three core prescriptions; S4: most important active ingredients in core prescription relevant to the target; S5: Venn map of the top 20 Reactome pathways in the core prescription; S6: forty high-degree targets from enrichment analysis based on the Kyoto Encyclopedia of Genes and Genomes pathway; S7: coacting genes in three core prescriptions; S8: sixteen high-degree hub genes linked with both rectal cancer and three core prescriptions; and S9: molecular docking results of active ingredients in core prescriptions. [file 1353674.f1.zip › 1353674.f1/S7 Co-acting genes in three core prescriptions.docx]
