# Supplementary material for: Data Mining, Network Pharmacology, and Molecular Docking Explore the Effects of Core Traditional Chinese Medicine Prescriptions in Patients with Rectal Cancer and Qi and Blood Deficiency Syndrome
Source: Evid Based Complement Alternat Med. 2021 Aug 2;2021:1353674. doi: 10.1155/2021/1353674 (PMC8360715; doi:10.1155/2021/1353674)
Supplement: Supplementary Materials — S1: top 20 herbs in three core prescriptions; S2: three core prescriptions; S3: core compounds with a common rank value > 200 in the three core prescriptions; S4: most important active ingredients in core prescription relevant to the target; S5: Venn map of the top 20 Reactome pathways in the core prescription; S6: forty high-degree targets from enrichment analysis based on the Kyoto Encyclopedia of Genes and Genomes pathway; S7: coacting genes in three core prescriptions; S8: sixteen high-degree hub genes linked with both rectal cancer and three core prescriptions; and S9: molecular docking results of active ingredients in core prescriptions. [file 1353674.f1.zip › 1353674.f1/S8 16 high degree hub genes linked with both recta.docx]

S8 16 high degree hub genes linked with both rectal cancer and three core prescriptions

| Gene | Degree(P1) | Degree(P2) | Degree(P3) | Inference Score |
| --- | --- | --- | --- | --- |
| AKT1 | 119 | 175 | 163 | 174.35 |
| IL6 | 115 | 173 | 164 | 183.88 |
| TP53 | 101 | 168 | 152 | 208.14 |
| TNF | 108 | 157 | 147 | 198.28 |
| CASP3 | 92 | 147 | 133 | 208.49 |
| VEGFA | 99 | 145 | 142 | 157.85 |
| MYC | 80 | 129 | 120 | 143.62 |
| ESR1 | 83 | 113 | 103 | 99.96 |
| PTGS2 | 83 | 109 | 103 | 231.43 |
| MMP9 | 76 | 109 | 107 | 149.37 |
| IL1B | 81 | 105 | 101 | 193.63 |
| IL10 | 72 | 103 | 100 | 137.93 |
| ERBB2 | 52 | 91 | 80 | 85.81 |
| MMP2 | 66 | 88 | 83 | 153.59 |
| PPARG | 71 | 85 | 79 | 165.77 |
| TGFB1 | 50 | 71 | 69 | 154.55 |
