# Supplementary material for: Data Mining, Network Pharmacology, and Molecular Docking Explore the Effects of Core Traditional Chinese Medicine Prescriptions in Patients with Rectal Cancer and Qi and Blood Deficiency Syndrome
Source: Evid Based Complement Alternat Med. 2021 Aug 2;2021:1353674. doi: 10.1155/2021/1353674 (PMC8360715; doi:10.1155/2021/1353674)
Supplement: Supplementary Materials — S1: top 20 herbs in three core prescriptions; S2: three core prescriptions; S3: core compounds with a common rank value > 200 in the three core prescriptions; S4: most important active ingredients in core prescription relevant to the target; S5: Venn map of the top 20 Reactome pathways in the core prescription; S6: forty high-degree targets from enrichment analysis based on the Kyoto Encyclopedia of Genes and Genomes pathway; S7: coacting genes in three core prescriptions; S8: sixteen high-degree hub genes linked with both rectal cancer and three core prescriptions; and S9: molecular docking results of active ingredients in core prescriptions. [file 1353674.f1.zip › 1353674.f1/S9 Molecular docking results of active ingredients.docx]

S9 Molecular docking results of active ingredients in core prescriptions.

| PubChem CID | ingredient | Energy(IL-6) | Energy(IL-10) |
| --- | --- | --- | --- |
| 985 | palmitic acid | -0.317511867 | -3.724796131 |
| 3893 | lauric acid | -2.745460625 | -6.998443982 |
| 3931 | linolenic acid | -0.51046095 | -5.108455006 |
| 33032 | Gulutamine | -3.261522042 | -4.713219132 |
| 8892 | hexanoic acid | -2.983708792 | -4.587896108 |
| 119 | gamma-aminobutyric acid | -2.746508038 | -3.695184809 |
| 305 | choline | -2.39323454 | -3.084162314 |
| 5280343 | quercetin | -1.713072393 | -4.745096618 |
| 5281 | stearic acid | -0.713017021 | -6.314688398 |
| 323 | coumarin | -4.286703772 | -5.227674977 |
| 145742 | Prolinum | -2.157229384 | -4.982400307 |
| 13849 | PENTADECYLIC ACID | -2.060685405 | -4.48113444 |
| 1110 | succinic acid | -0.920237738 | -6.17784649 |
| 5950 | LPG, L-Alanine | -2.602297842 | -5.318465592 |
| 5280863 | kaempferol | -2.745460625 | -6.998443982 |

S9 Molecular docking results of active ingredients in core prescriptions.

| PubChem CID | ingredients | Energy  (IL1B) | Energy  (TGFB1) | Energy  (PPARG) | Energy  (MMP9) | Energy  (MYC) |
| --- | --- | --- | --- | --- | --- | --- |
| 985 | palmitic acid | -2.285728271 | -3.988387714 | -5.736662135 | -4.167845958 | -2.392589838 |
| 3893 | lauric acid | -2.618242834 | -7.460383909 | -8.111559126 | -6.892702339 | -5.324790662 |
| 3931 | linolenic acid | -2.203134687 | -5.305195939 | -6.501537575 | -4.435809889 | -3.489771006 |
| 33032 | Gulutamine | -2.406479107 | -4.308710466 | -4.601745276 | -3.96622032 | -3.422129908 |
| 8892 | hexanoic acid | -2.235467622 | -5.376442591 | -5.795913075 | -4.576213703 | -3.240463364 |
| 119 | gamma-aminobutyric acid | -1.96362037 | -3.459416982 | -4.129758169 | -3.660039689 | -2.65386231 |
| 305 | choline | -1.950045566 | -2.925239644 | -2.963528396 | -3.090225059 | -2.349488481 |
| 5280343 | quercetin | -1.596922178 | -4.142946502 | -6.192491689 | -4.070609114 | -3.628297149 |
| 5281 | stearic acid | -2.923663163 | -6.435378938 | -4.522150014 | -6.637112198 | -4.480388638 |
| 323 | coumarin | -3.639640658 | -5.204947895 | -6.581586038 | -4.978195376 | -4.116159259 |
| 145742 | Prolinum | -1.628192293 | -4.734932765 | -6.355384427 | -4.105197379 | -2.680246628 |
| 13849 | PENTADECYLIC ACID | -2.03633554 | -5.155287673 | -5.761152268 | -4.077707627 | -3.137808281 |
| 1110 | succinic acid | -2.31211375 | -6.974898534 | -5.649409739 | -5.868368072 | -4.335733484 |
| 5950 | LPG, L-Alanine | -2.579955953 | -5.316144303 | -6.711766809 | -4.484425866 | -2.730374834 |
| 5280863 | kaempferol | -2.618242834 | -7.460383909 | -8.111559126 | -6.892702339 | -5.324790662 |
